# Chronic Abdominal Pain and Gastrointestinal Symptoms 12 Years After Primary Roux-en-Y Gastric Bypass: A Cross-Sectional Controlled Study

**DOI:** 10.1007/s11695-025-07933-2

**Published:** 2025-06-04

**Authors:** Åsne Ask Hyldmo, Dag Arne Lihaug Hoff, Arne Wibe, Kirsti Kverndokk Bjerkan, Siren Nymo, Gjermund Johnsen, Hallvard Græslie, Jorunn Sandvik

**Affiliations:** 1 Department of Clinical Studies, Møre and Romsdal Hospital Trust, Ålesund, Norway; 2 Department of Clinical and Molecular Medicine, Faculty of Medicine and Health Sciences, Norwegian University of Science and Technology, Trondheim, Norway; 3 Department of Health Sciences, Faculty of Medicine and Health Sciences, Norwegian University of Science and Technology, Ålesund, Norway; 4 Department of Surgery, Ålesund Hospital, Møre and Romsdal Hospital Trust, Ålesund, Norway; 5 Clinic of Surgery, Namsos Hospital, Nord-Trøndelag Hospital Trust, Namsos, Norway; 6 Institute of Public Health and Nursing, Faculty of Medicine and Health Sciences, Norwegian University of Science and Technology (NTNU), Trondheim, Norway; 7 National Research Center for Minimally Invasive and Image Guided Diagnostics and Therapy, Center for Innovation, Medical Equipment and Technology, St. Olav`s hospital, Trondheim University Hospital, Trondheim, Norway; 8 Centre for Obesity Research, Clinic of Surgery, St. Olav`s hospital, Trondheim University Hospital, Trondheim, Norway

**Keywords:** Bariatric surgery, Roux-en-Y gastric bypass, RYGB, Chronic abdominal pain, Gastrointestinal symptoms, General population, The HUNT4 study

## Abstract

**Background:**

The knowledge on chronic abdominal pain and gastrointestinal (GI) symptoms as a long-term complication to Roux-en-Y gastric bypass (RYGB) is limited. The aim of this study was to explore the frequency and characteristics of chronic abdominal pain and GI symptoms long-term after primary RYGB compared to the general population.

**Methods:**

A cross-sectional study of patients 10–15 years after RYGB compared to a control group with a similar age and sex distribution from the HUNT4 Survey. Abdominal pain and GI symptoms were assessed with a self-reported questionnaire.

**Results:**

A total of 496 patients were included, with a mean observation time of 11.7 ± SD 1.6 years after RYGB. The controls were 3000 participants from HUNT4. Chronic abdominal pain was reported by 162 (32.7%) RYGB patients compared to 697 (23.2%) of the controls (*p* < 0.001). Bloating was the most common GI symptom reported by 356 (72%) RYGB patients. Eighty-two (16.5%) RYGB patients and 302 (10.1%) controls graded chronic pain as severe (*p* < 0.001). Severe chronic pain after RYGB was more often located in the upper part of the abdomen in 58 (71%) patients, associated with severe nausea in 27 (33%) patients, and less often relieved after defecation in 35 (42%) patients compared to the controls (*p* < 0.001).

**Conclusions:**

Twelve years after RYGB, a third of patients reported chronic abdominal pain compared with a quarter of controls. Half of the participants with chronic abdominal pain in both groups rated the pain as severe. The location of severe pain and associations with other gastrointestinal symptoms differed between the two groups.

**Graphical Abstract:**

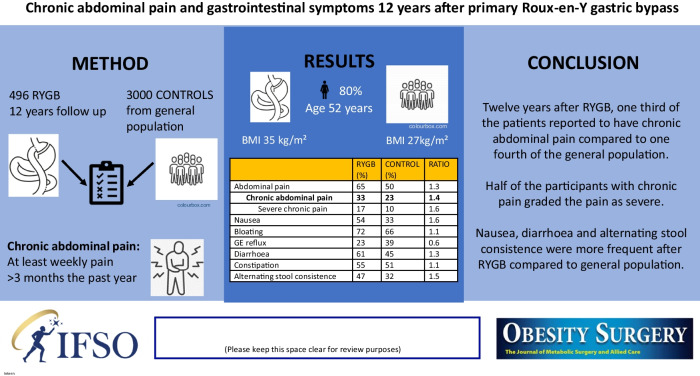

## Introduction

Metabolic bariatric surgery (MBS) is the most effective treatment for severe obesity, with a significant and durable weight loss, in addition to remission of obesity-related diseases and prolonged survival [1–4]. Roux-en-Y gastric bypass (RYGB) is the second most common MBS procedure, accounting for 28.5% of the primary procedures and 59.7% of the revision procedures worldwide [5]. Although general health improves for the majority of patients after RYGB, many of them will experience adverse side effects. Abdominal pain is one of the most frequent reasons for RYGB patients to contact healthcare professionals [6]. Despite thorough examinations, the cause of the pain remains unclear in a third of the patients [7–9].

The International Associations for the Study of Pain (IASP) defines pain as “an unpleasant sensory and emotional experience associated with or resembling that associated with actual or potential tissue damage” [10]. Furthermore, the IASP defines pain as chronic when it lasts or recurs for more than 3 months. They also advocate that chronic pain optionally should be classified according to severity [11]. Epidemiological data estimates the global prevalence of abdominal pain in the last 3 months to be about 50% [12], and chronic abdominal pain is one of the most frequent reasons for visits in general practice [13].

The frequency of chronic abdominal pain up to 5 years after RYGB is reported to be 7–54% and varies due to different definitions and methods used to report symptoms [6, 7, 14–18]. Previous studies have reported a higher frequency of gastrointestinal (GI) symptoms after RYGB compared to controls without MBS, as well as increased GI symptom scores in patients 2 years after RYGB [19, 20]. A cross-sectional study of patients 3 years after MBS reported similar GI symptom scores after RYGB and sleeve gastrectomy (SG), but a higher frequency of abdominal pain after RYGB [21].

As RYGB induces anatomical and physiological alterations, chronic abdominal pain and GI symptoms may be related to surgical complications, gallstone disease, unfavourable eating habits, and altered gut-brain interaction [22, 23]. “Disorders of gut-brain interaction” has replaced the term “Functional gastrointestinal disorders” and is defined by the ROME foundation as “A group of disorders classified by GI symptoms related to any combination of motility disturbances, visceral hypersensitivity, altered mucosal and immune function, gut microbiota, and/or central nervous system processing” [24]. One of these disorders is irritable bowel syndrome (IBS), characterized by chronic abdominal pain associated with a change in stool form or frequency [25]. IBS affects between 4 and 10% of the general population dependent on the criteria used [25, 26]. The prevalence of IBS-like symptoms is elevated in patients with obesity [27] and has been reported to increase after RYGB [28].

Knowledge on chronic abdominal pain and GI symptoms as a long-term complication to RYGB is limited [29]. The aim of this study was therefore to explore the frequency and characteristics of self-reported chronic abdominal pain and GI symptoms in patients 10–15 years after RYGB compared to the general population.

## Methods

### Study Setting and Design

The Bariatric Surgery Observation Study (BAROBS) is a cross-sectional observational study supplemented by data from the electronic patient records of patients who underwent primary RYGB at three public hospitals in the Central Norway Regional Health Authority during 2003–2009. The Barobs study included a clinical follow-up with an interview, anthropometric measurements, and a self-reported questionnaire during 2018–2020. Criteria for bariatric surgery at that time were body mass index (BMI) ≥ 40 kg/m^2^ or BMI ≥ 35 kg/m^2^ with obesity-related comorbidity and an age from 18 to 60 years. The RYGB procedure was performed laparoscopically with a gastric pouch of 30–50 ml, a biliopancreatic limb of 40–60 cm, and an ante-colic alimentary limb of 100 cm (preoperative BMI < 50 kg/m^2^) or 150 cm (preoperative BMI ≥ 50 kg/m^2^). The mesenteric defects were not closed at the initial operations [30].

The control group consisted of randomly selected participants with a similar sex distribution and mean age from the fourth survey of the Trøndelag Health Study (HUNT4), representing the general population. The Trøndelag Health Study is a collaboration between HUNT Research Centre (Faculty of Medicine and Health Sciences, Norwegian University of Science and Technology NTNU), Trøndelag County Council, Central Norway Regional Health Authority, and the Norwegian Institute of Public Health [31].

### Measurements and Questionnaires

Weight and height were measured at attendance in both studies. Medical information from the time of RYGB surgery and follow-up programme, including history of abdominal surgery after RYGB, was collected from the electronic patient record. Information on abdominal surgical history was not available for the control group. The questions from the self-reported questionnaires in BAROBS and HUNT used in this study were identical and included socioeconomics, smoking status, use of medications, general health, and GI symptoms such as abdominal pain, nausea, reflux/heartburn, bloating, diarrhoea, constipation, and alternating constipation and diarrhoea. Questions on abdominal pain were based on the ROME II criteria for IBS, as in HUNT3 and HUNT4, and included frequency, severity, and location of pain in addition to relations to food intake, defecation, stool frequency, or changes in stool consistency (more soft or more solid). The current ROME IV criteria were not available at the time of HUNT3. The answer options for the questions on abdominal pain and other GI symptoms were graded according to severity with “never”, “moderate”, or “severe”.

### Definition

Chronic abdominal pain was defined as at least weekly pain with a duration of more than 3 months in the last year. Sub-analyses of the participants who reported severe chronic abdominal pain were made to explore associations between the severe pain, characteristics of pain, other GI symptoms, and independent risk factors.

### Statistical Analyses

Categorical variables are reported as numbers and percentages and compared by the Pearson chi-square test. All continuous variables were normally distributed and reported as means with standard deviations (SD). The continuous variables were analysed using the independent sample *t*-test. The risk of severe chronic abdominal pain versus no pain was analysed by a multivariate logistic regression based on forward selection of univariate analyses of sex, age, BMI, smoking, comorbidities, and abdominal surgery during the observation time. Results are reported as odds ratios (OR) with 95% confidence intervals (95% CI). *P* < 0.05 was considered significant for all statistical analyses. Multiple imputation in SPSS was performed for missing data from the self-reported questionnaire. The statistical analyses were performed by using IBM SPSS Statistics version 29.0.1. (SPSS Inc., Chicago, IL, USA).

## Results

Out of 959 patients who underwent RYGB in the defined period, 546 (57%) participated in the BAROBS study. After exclusion of 20 patients who had RYGB as a secondary procedure and 30 patients with missing data on abdominal pain, a total of 496 patients were included in the analyses. The mean ± SD observation time after RYGB was 11.7 ± 1.6 years, age at follow-up was 51.3 ± 8.8 years, BMI was 34.9 ± 7.0 kg/m^2^, and 393 (79.2%) were women.

The control group consisted of 3000 persons with similar sex distribution and mean age as the RYGB group, with BMI 27.3 ± 4.8 kg/m^2^ (Table 1). Any degree of abdominal pain was reported by 324 (65.3%) patients and chronic abdominal pain by 162 (32.7%) patients in the RYGB group, compared to 1492 (49.7%) and 697 (23.2%) of the controls, respectively (*p* < 0.001). Chronic abdominal pain was graded as severe in 82 (16.5%) RYGB patients and in 302 (10.1%) of the controls (*p* < 0.001). Nausea and diarrhoea were more common in RYGB patients, with frequencies of 270 (54.4%) and 300 (60.5%) versus 1003 (33.4%) and 1346 (44.9%) of the controls. Gastroesophageal (GE) reflux was less frequent after RYGB, with 112 (22.6%) patients compared to 1166 (38.9%) in the control group (*p* < 0.001 for all) (Table 2).

**Table 1:** Characteristics of the participants in the RYGB group and the control group

	RYGB (*n* = 496)	Control (*n* = 3000)	***p***
Female	393 (79.2)	2400 (80.0)	
Age, years			
At RYGB	39.6 (8.7)	N/A	
At data collection	51.3 ± 8.8	52.1 ± 9.4	
Observation time after RYGB, years	11.7 ± 1.6	N/A	
BMI kg/m^2^			
Before RYGB	46.5 ± 5.5	N/A	
At data collection	34.9 ± 7.0	27.3 ± 4.8	** < 0.001**^**b**^
TWL, %			
From before RYGB to nadir	36.3 (8.0)	N/A	
From before RYGB to data collection	25.4 (11.4)	N/A	
Education, years			** < 0.001**^**a**^
≤ 10	49 (9.9)	153 (5.1)	
10–13	287 (57.9)	1385 (46.2)	
≥ 13	160 (32.3)	1462 (48.7)	
Income per year, EURO			** < 0.001**^**a**^
≤ 37,800	171 (34.5)	585 (19.5)	
37,800–84,000	261 (52.6)	1660 (55.3)	
≥ 84,000	64 (12.9)	755 (25.2)	
Current smoking	129 (26.0)	338 (11.3)	** < 0.001**^**a**^
Comorbidities			
T2DM	86 (17.3)	136 (4.5)	** < 0.001**^**a**^
Hypertension	119 (24.0)	453 (15.1)	** < 0.001**^**a**^
Dyslipidaemia	41 (8.1)	293 (9.8)	0.290^a^
Anxiety/depression	90 (18.1)	199 (6.6)	** < 0.001**^**a**^
Medication for GE reflux	47 (9.5)	310 (10.3)	0.521
Medication for constipation	65 (13.1)	160 (5.3)	** < 0.001**^**a**^
Abdominal surgery after RYGB	207 (41.7)	N/A	
Cholecystectomy	70 (14.1)		
Internal herniation	53 (10.7)		

*RYGB* Roux-en-Y gastric bypass, BMI body mass index, TWL total weight loss, T2DM diabetes mellitus type II, GE gastroesophageal, N/A not applicable

BMI and TWL% were calculated based on the weight and height measured before RYGB, at nadir 12–24 months after RYGB, and at follow-up. Values are presented as mean ± SD or number (%). ^a^*P*-value of Pearson chi-square test. ^b^*P*-value for independent sample *t*-test

**Table 2:** Abdominal pain and gastrointestinal symptoms in the RYGB group and the control group

	RYGB, (*n* = 496) (%)	Control (*n* = 3000) (%)	***p***
Abdominal pain			
Never	172 (34.7)	1508 (50.3)	
Overall	324 (65.3)	1492 (49.7)	
At least weekly frequency	162 (32.7)	697 (23.2)	**< 0.001**
Moderate	80 (16.1)	395 (13.2)	
Severe	82 (16.5)	302 (10.1)	**< 0.001**
Nausea			**< 0.001**
Never	226 (45.5)	1997 (66.6)	
Moderate	225 (45.4)	941 (31.3)	
Severe	45 (9.1)	62 (2.1)	
Bloating			**< 0.001**
Never	140 (28.2)	1016 (33.9)	
Moderate	212 (42.7)	1436 (47.9)	
Severe	144 (29.0)	548 (18.3)	
GE reflux/heartburn			**< 0.001**
Never	384 (77.4)	1834 (61.1)	
Moderate	82 (16.5)	961 (32.0)	
Severe	30 (6.1)	205 (6.9)	
Diarrhoea			**< 0.001**
Never	196 (39.5)	1654 (55.1)	
Moderate	229 (46.2)	1183 (39.4)	
Severe	71 (14.3)	163 (5.5)	
Constipation			0.102
Never	224 (45.1)	1480 (49.3)	
Moderate	218 (44.0)	1264 (42.1)	
Severe	54 (10.9)	256 (8.6)	
Alternating stool consistence			**< 0.001**
Never	263 (53.0)	2038 (67.9)	
Moderate	189 (38.1)	827 (27.6)	
Severe	44 (8.9)	135 (4.5)	

Data presented in numbers and percentages. *P*-value for Pearson chi-square test

In the sub-analysis of participants with severe chronic abdominal pain, the pain was located in the upper abdomen in 58 (70.7%) RYGB patients versus 144 (47.7%) of the controls (*p* < 0.001). A pain-relieving effect after defecation was reported in 35 (42.7%) of the RYGB patients compared to 190 (62.9%) of the controls (*p* < 0.001). There was no difference in the increase of pain after meals between the two groups (Table 3). Furthermore, severe nausea was reported by 27 (32.9%) RYGB patients compared to 32 (10.6%) of the controls (*p* < 0.001) (Table 4).

**Table 3:** Characteristics of severe chronic abdominal pain in the RYGB group and the control group

	RYGB (*n* = 82) (%)	Control (*n* = 302) (%)	***p***
Pain localized in upper abdomen	58 (70.7)	144 (47.7)	***p* < 0.001**
Pain worsening after meals	44 (53.7)	167 (55.3)	*p* = 0.791
Pain relieved after defecation	35 (42.7)	190 (62.9)	***p* < 0.001**
Pain associated with defecation frequency	24 (29.3)	133 (44.0)	***p* = 0.016**
Pain associated with change in stool consistence (more soft or more solid)	29 (35.4)	166 (55.0)	***p* = 0.002**

Data presented in numbers and percentage. *P*-value for Pearson chi-square test

**Table 4:** Gastrointestinal symptoms in participants with severe chronic abdominal pain

	**RYGB (***n* = 82) (%)	**Control** (*n* = 302) (%)	***p***
Nausea			** < 0.001**
Never	16 (19.5)	119 (39.4)	
Moderate	39 (47.6)	151 (50.0)	
Severe	27 (32.9)	32 (10.6)	
Bloating			0.067
Never	10 (12.2)	17 (5.6)	
Moderate	18 (22.0)	93 (30.8)	
Severe	54 (65.9)	192 (63.6)	
GE reflux/heartburn			**0.036**
Never	49 (59.7)	132 (43.7)	
Moderate	20 (24.4)	106 (35.1)	
Severe	13 (15.9)	64 (21.2)	
Diarrhoea			0.954
Never	18 (21.9)	69 (22.8)	
Moderate	38 (46.3)	144 (47.7)	
Severe	26 (31.7)	89 (29.5)	
Constipation			0.941
Never	23 (28.0)	87 (28.8)	
Moderate	37 (45.1)	139 (46.0)	
Severe	22 (26.8)	76 (25.2)	
Alternating stool consistence			0.816
Never	28 (34.1)	114 (37.8)	
Moderate	36 (43.9)	123 (40.7)	
Severe	18 (22.0)	65 (21.5)	

Data presented in numbers and percentage. *P*-value for Pearson chi-square test

Fifty-seven (69.5%) of the 82 RYGB patients who suffered from severe abdominal pain underwent abdominal surgery during the observation period, of whom 27 (32.9%) underwent cholecystectomy and 22 (26.8%) surgery for internal herniation. Women had an independent risk of severe chronic pain in both groups (OR 3.79, 95%CI 1.56, 9.24 after RYGB and OR 1.98, 95%CI 1.38, 2.85 in the controls). In the RYGB group, undergoing abdominal surgery after RYGB increased the risk (OR 3.82, 95%CI 2.21, 6.60), while increasing BMI (kg/m^2^) reduced the risk of severe chronic pain (OR 0.95, of the controls had pain (OR 2.09, 95%CI 1.40, 3.13) (see Table 5 for further details).

**Table 5:** Multiple logistic regression for risk factors for predicting severe chronic abdominal pain versus no pain in the RYGB group and in the control group

	RYGB (*n* = 402)				Control (*n* = 2559)			
	Univariate OR (95%CI)	*p*-value	Multivariate adjusted OR (95% CI)	*p*-value	Univariate OR (95%CI)	*p*-value	Multivariate adjusted OR (95% CI)	*p*-value
Sex, female	4.58 (1.93, 10.91)	< 0.001	3.79 (1.56, 9.24)	**0.003**	2.05 (1.43, 2.95)	< 0.001	1.98 (1.38, 2.85)	** < 0.001**
Age, year	0.99 (0.96, 1.02)	0.420			0.99 (0.99, 1.00)	0.144		
BMI, kg/m^2^	0.92 (0.89, 0.96)	< 0.001	0.95 (0.91, 0.99)	**0.013**	1.02 (1.00, 1.05)	0.063		
Current smoking	1.13 (0.66, 1.93)	0.665			1.32 (0.92, 1.88)	0.127		
T2DM	1.26 (0.67, 2.39)	0.472			1.23 (0.72, 2.12)	0.451		
Hypertension	0.95 (0.54, 1.66)	0.850			1.00 (0.71, 1.40)	0.981		
Dyslipidaemia	1.33 (0.60, 2.96)	0.480			1.10 (0.74, 1.63)	0.632		
Anxiety/depression	1.60 (0.90, 2.84)	0.109			2.23 (1.49, 3.32)	< 0.001	2.09 (1.40, 3.13)	** < 0.001**
Abdominal surgery after RYGB	4.53 (2.67, 7.70)	< 0.001	3.82 (2.21, 6.60)	** < 0.001**	N/A			

## Discussion

The main finding of this study was that 12 years after RYGB, two-thirds of the patients reported having some degree of abdominal pain, compared to half of the controls from the general population with similar age and gender distribution. Moreover, one in three RYGB patients reported having chronic abdominal pain compared to one in four of the controls, and approximately half of the participants with chronic abdominal pain in both groups graded the pain as severe. This confirms results from previous studies. In a Danish cross-sectional study of 1429 patients 5 years after RYGB, almost 55% reported abdominal pain and one third of them had received health care because of abdominal pain [6]. Studies that have defined chronic pain according to the IASP have reported rates of chronic abdominal pain after RYGB from 7 to 34%. The wide range may be due to different study designs or observation times after RYGB, but none of them had an observation period of more than 5 years [11, 14, 15, 18].

In the present study, one out of six RYGB patients graded the chronic pain as severe. The IASP has recommended for the 11 th edition of the International Classification of Diseases (ICD 11) that pain optionally should be classified according to severity and has the severity of chronic pain as “a compound of pain intensity, pain-related distress and task interference”[11]. In a study of 787 RYGB patients with 5-year observation time, Gormsen et al. used two definitions of chronic abdominal pain: patients with self-reported pain more than twice a week for more than 3 months and patients with a pain diagnosis from a specialized pain clinic or who had a prescription for strong painkillers. The prevalence was 21% and 11%, respectively, but with overlap for the two conditions [11]. Recurrent abdominal pain for more than twice a week indicates indeed a chronic health problem but does not include the severity of the pain. However, the use of strong painkillers or being referred to a specialized pain clinic is only indicated for patients with severe pain conditions and represents a stricter definition than that used in our study.

When it comes to other GI symptoms, we found a higher frequency of reported bloating, nausea, diarrhoea, and alternating stool consistency among the RYGB patients, with bloating as the most frequent symptom. GE reflux was more prevalent in the controls, while constipation did not differ between the groups. In previous studies, frequencies of gastrointestinal complaints and IBS-like symptoms are reported to increase after RYGB and to be higher after RYGB compared to controls with obesity [19, 20, 28]. However, in the present study, the RYGB patients with severe chronic abdominal pain reported less association with change in stool form or frequency compared to that in the general population. This suggests that the severe chronic pain after RYGB differs from the pain of IBS, which is associated with change in stool form or frequency [25].

Severe nausea was reported more frequently in the RYGB patients and could be related to several physiological changes following RYGB, like changes in neural responses or gut peptides, in addition to maladaptive eating habits, early satiety, or the dumping syndrome [20, 32]. Since nausea is reported to be one of the most common side effects of GLP1 analogues, nausea may also be a symptom of high GLP1 levels in selected individuals after RYGB [33, 34].

In seven out of ten of the RYGB patients, the pain was located in the upper abdomen compared to half of the controls. Alternatively, this may also be an expression of maladaptive eating habits or food intolerance after RYGB [20]; on the other hand, worsening pain after meals did not differ between the groups.

To undergo abdominal surgery after RYGB increased the risk of having chronic severe abdominal pain, consistent with previously published findings on cholecystectomies in the BAROBS study. The authors reported that 12% of the patients had undergone cholecystectomy prior to RYGB, 16% underwent cholecystectomy during the observation time of 11.5 years, and a history of cholecystectomy was associated with chronic abdominal pain [35]. A previous publication from the HUNT study reported a frequency of cholecystectomies of 1.8% during an observation time of 15 years [36]. Women had an increased risk of abdominal pain in both groups, consistent with previous studies [37].

## Strengths and limitations

To our knowledge, this is the first study to compare abdominal pain more than a decade after RYGB with a control group representing the general population. Despite a follow-up rate of 57%, a study population of 546 participants is large a decade after RYGB.

The groups were similar with regard to mean age and sex distribution, with the aim of comparing a common health complaint after RYGB with the general population. However, the difference in morbidity and socioeconomic status between the two groups may have influenced the results. A limitation is the lack of information on abdominal surgical history for the control group. Even though the control group represented a population with a similar prevalence of abdominal surgeries as the HUNT population in general, the surgical history of the two groups at baseline was not directly comparable.

The study had a cross-sectional design, not suitable for investigating longitudinal development and causal relationships. Although data from the patients’ medical records were available for the RYGB group, data on GI symptoms before and shortly after RYGB was not recorded.

## Conclusion

Twelve years after RYGB, one third of the patients reported chronic abdominal pain compared to one fourth of the general population. Almost half of the participants with chronic abdominal pain in both groups graded the pain as severe. GI symptoms, like bloating, nausea, diarrhoea and alternating stool consistency, were more often reported by the RYGB patients. The location of severe pain and associations with other gastrointestinal symptoms differed between the two groups.

## Data Availability

No datasets were generated or analysed during the current study.
